# Application of Nonsurgical Modalities in Improving Facial Aging

**DOI:** 10.1155/2022/8332631

**Published:** 2022-02-24

**Authors:** Kelun Li, Fanyu Meng, Yu Ru Li, Yueyi Tian, Hao Chen, Qi Jia, HongXin Cai, Heng Bo Jiang

**Affiliations:** ^1^The Conversationalist Club, School of Stomatology, Shandong First Medical University and Shandong Academy of Medical Sciences, Tai'an, Shandong 271036, China; ^2^Department of Dental Biomaterials and Bioengineering, Yonsei University College of Dentistry, Seoul 03722, Republic of Korea

## Abstract

**Objective:**

This review aims to summarize different kinds of applications of minimally invasive surgery in improving facial aging to provide a comprehensive and accurate introduction on the issue of esthetic treatment of facial skin. *Overview*. In the twentieth century, facial rejuvenation has become a new beauty trend. Facial cosmetology has entered a period of antiaging and rejuvenation therapies and microplastic surgery. The pursuit of beauty has promoted the development of minimally invasive plastic surgery. This review introduces the possible causes of facial aging and its related topics with a focus on facial injectable drugs, such as botulinum toxin, main filler materials (hyaluronic acid, calcium hydroxyapatite, poly L-lactic acid, collagen, autologous fat, and polymethyl methacrylate), and some current antiwrinkle technologies, such as thread lift and radiofrequency rhytidectomy.

**Conclusions:**

Despite the difference in mechanisms of action, each technique can address facial aging involving the loss of collagen, displacement and enlargement of fat, and muscle relaxation. Combinations of these treatments can provide patients with reasonable, comprehensive, and personalized treatment plans.

## 1. Introduction

Beauty is an eternal theme for humans as we tend to increasingly focus on their appearances and hope to stay younger as they age. Facial plastic surgery improves facial appearance and function and has been practiced since more than 100 years ago. Jacques Joseph is considered the father of modern facial plastic surgery, and he developed many of the earliest surgical technologies. In recent years, minimally invasive facial beauty has evolved rapidly. In the past two decades, the use of injectable botulinum toxin has increased by 621% [1]. Compared with traditional facial cosmetic surgery, nonsurgical modalities results in more natural, nonstiff, and smoother results with very small trauma and significantly shorter recovery, therefore its preference by patients [2]. This technique is directed toward adjusting the various details of the face, such as wrinkles and fine lines, asymmetry, and excess fat [3]. In recent years, many novel, nonsurgical, antiregional aging treatments have emerged to address the signs of aging. This study analyzes the main reasons and good hair part of facial aging and introduces several hotter minimally invasive wrinkles: injection filler technology, including facial injections, several major facial injection filler materials, linear conduct, radiofrequency rhytidectomy, and other methods. Their mechanisms of action, indications, contraindications, complications, advantages, and disadvantages are discussed.

## 2. Aging of the Face

### 2.1. Possible Causes of Facial Aging

#### 2.1.1. Skin

External environmental factors and the body's internal factors can affect the aging process of facial skin. Indoor and outdoor air pollution [4, 5], such as skin exposure to soot, can lead to more obvious pigmentation spots and wrinkles. Sunlight and smoking can also accelerate the aging of the skin, which results in weakness, thinning, and wrinkling [5, 6]. The elasticity of the skin primarily depends on the dermis with collagen playing the primary role. With internal aging of the skin, the synthesis rate of collagen decreases and its degradation rate increases [7]. Moreover, the decomposition of collagen was increased by upregulating protease [8]. By the age of 40–50 years, the biosynthesis of elastin begins to decline sharply. Elastin is lost via natural degradation [9], which results in the loss of skin elasticity. Glycosaminoglycans play a crucial role in the absorption of water by the skin. With the degradation of hygroscopic glycosaminoglycans, the moisture in the skin is also gradually lost. Additionally, poor quality of sleep can also affect skin quality [10].

#### 2.1.2. Fat

**Figure 1:**
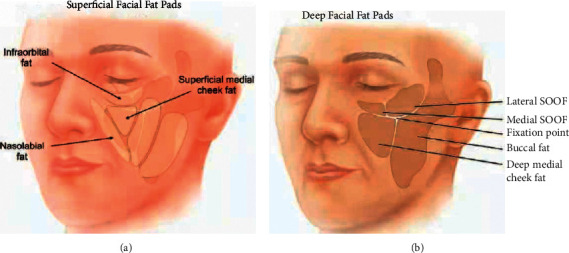
(a) The superficial fat (light beige) in the middle of the face includes the superficial fat on the inner side of the cheek, the fat in the nasolabial groove, and the fat within the orbit. (b) The midface deep fat (dark tan) compartment consists of the deep fat compartment of the inner cheek and the inner part of the buccal fat pad [11].

Facial fat is divided into superficial fat and deep fat by the superficial fascia system (Figure 1) [11]. Both shallow and deep fat tissues are separated into multiple smaller pieces of fat [12]. Fibrous tissue supports the fat in all parts. With the increase of age, the change of fat interval position leads to the change of adipose tissue contour [13]. Generally, these changes include atrophy of the deep fat tissue and displacement and hypertrophy of the shallow fat tissue [11, 13]. The continuous pressure of the deep layer of fat tissue on the bones and its relative inertia as a space-filling interface may explain the tendency of its selective atrophy with time [11, 14].

The shallow fat tissue of the forehead and the orbital and perioral fat also undergo atrophy [15]. The formation of facial wrinkles is also related to a reduction in the volume of white adipose tissue [16]. The reduction in subcutaneous fat leads to the relaxation of the skin around the nose, orbit, and chin and the formation of wrinkles [8]. The volume of the lower part of the nasolabial fat tissue increases, which results in the protrusion of the nasolabial groove, maxilla, and mandible of the inferior margin of the orbicularis oculi muscle and the deepening of the nasopharyngeal folds are the results of the downward displacement and volume loss of the buccal fat cavity [17]. The volume loss of the nasolabial sulcus and the head of the medial buccal adipose septum may also lead to lacrimal groove deformities, which aggravates facial aging and makes the nasopharyngeal folds and palpebral sulcus more obvious [18]. The loss of volume results in a lack of support for the medial and middle chambers of the cheek fat, thus resulting in a downward displacement of the facial septum, which is also an important factor in the development of wrinkles [19]. Gravity can also cause downward movement of the facial fat [20]. Due to the separation of fat parts, the change of fat content will be shown in the corresponding facial areas [21].

#### 2.1.3. Muscle

Facial muscles can be divided into expressive and masticatory muscles. The formation of facial wrinkles is mainly due to the relaxation of the superficial expressive muscles located in the superficial myofascial system, which connects the skin with the fat septum [8], which causes sagging of the skin and the formation of wrinkles. Muscle aging mainly results from changes in muscle tension and repetitive movements of the muscle [22]. Specifically, as age increases, the facial muscles lengthen, the muscle tension increases, the motion amplitude shortens, and the muscle tension at rest is closer to the maximum contraction tension [23]. However, facial muscle exercises have limited effects in restoring muscle tension [24]. In addition, the changes in facial muscles may also be a result of adaptation in response to the changes in facial ligaments and bones [13].

#### 2.1.4. Bone

Facial bones support the facial soft tissues. In the aging of facial bones, the most obvious change is the change in the mandibular angle from an L-shape to an I-shape, which leads to mandibular protrusion and changes in the mandibular line [7]. In addition, the zygomatic arch undergoes anteroposterior reconstruction, thus deepening the zygomatic fossa. After 30 years of age, there may be pit regression [25] and maxillary retrusion, which may lead to flat cheeks as well as depression and widening of the upper lip [26].

Between the ages of 30 and 50 years, the lower forehead may begin to flatten out as the angle between the eyebrows decreases. Additionally, there may be drooping of the nasal tip and widening of the alar base [27]. On the right side, these changes cause the face to rotate clockwise relative to the skull base: the brow, orbit, piriform hole, and maxilla rotate downward, thus resulting in a flattened facial angle [28, 29]. The nasal cavity expands outward and forward, and the upper and olfactory cavities remain intact, thus resulting in the enlargement of the piriform foramen. The rim of the eye socket expands downward and outward, thus causing the socket to lose its roundness, and the chin becomes more protrusive, oblique, and short [7].

### 2.2. Areas of Aging

There are several signs of aging on the face. Of them, the more obvious and significant ones include the appearance of a vertical line in the interbrow and the atrophy of the upper cheeks or midface and the nasolabial folds. Other more common signs of facial aging include horizontal forehead creases, temporal or brow hollows, tear troughs, nasal wrinkles, vertical lip lines, thinning lips, irregular jawline, atrophy of the preauricular fat, and thinning earlobes [30, 31].

The upper face consists of the forehead, interbrow, ocular, and temporal regions. The aging of the upper face is mainly reflected on the forehead [32]. The skin of the forehead loses its elasticity over time, resulting in an irregular and wrinkled forehead with changes in its color [33, 34]. The lower third of the forehead also appears slightly concave due to the formation of more wrinkles on the forehead and plateauing between the eyebrows [35]. Although it is fixed by facial loose tissue, the interval of facial loose tissue will shrink and decrease over time, resulting in skin wrinkling [36]. In this area, a combination of fillers and botulinum toxin injections will be more effective than fillers alone.

The middle of the face refers to the area between the lower eyelid and corners of the mouth where fillers are most commonly used [37]. Volume loss of the soft tissues is an essential aspect of facial aging [38]. Aging of the midface leads to an overall drooping of the soft tissues, particularly the orbicularis oculi and the soft tissue of the zygomatic complex. The decline in these tissues causes, in part, a widening in the laughter line or the nasolabial groove [26]. With aging, the orbital width increases, and the contours of the eyes gradually change [39]. The reduction of fat in this area can also be a cause of thinning of the temples, which results in an emaciated look [40].

The lower face is the area between the corners of the mouth and the neck. Most symptoms of aging in the lower face, such as perioral wrinkles, are also caused by atrophy of the soft tissues and bones. Perioral wrinkles are more commonly treated with fillers to slow the signs of aging. Volume fillers in the lips are also very common [41, 42]. In the jaw and chin, chin augmentation and filling of the anterior jawline are common procedures [43]. However, studies have demonstrated that chin deformities can result when the jaw's soft tissue drops to overfill the chin [44].

## 3. Facial Injections

### 3.1. Botulinum Toxin

Botulinum toxin, like other natural substances such as atropine and paclitaxel, was first recognized for its toxicological effects and was used as a powerful biological weapon 70 years ago [45]. It was first used to treat strabismus in the 1970s [46] and was finally approved for human use by the US Food and Drugs Safety Administration (FDA) in 1989.

Facial expressions: muscle contractions move the skin over the face and create wrinkles and folds perpendicular to the shape of the muscles. Obligate anaerobic *Clostridium botulinum* can produce seven serotypes of botulinum toxin (A, B, C, D, E, and F); of them, type A is the most toxic and commonly used (Table 1) [48]. Although different serotypes of botulinum toxin have unique biochemical properties, all have a semblable mechanism of action: they cause muscle paralysis and relaxation by blocking cholinergic nerve transmission. This mechanism can be used to reduce the hyperactivity of muscles of expression, thus improving and eliminating wrinkles. The effect is persistent but reversible to some extent [49]. The formation of SNARE protein is necessary for the binding of acetylcholine to the presynaptic membrane as well as the release of acetylcholine. Botulinum toxin cleaves the substrate of this protein, thus inhibiting the release of acetylcholine. Over time, the effects of botulinum toxin decrease with the formation of new axons and motor endplates [50].

#### 3.1.1. Indications

**Figure 2:**
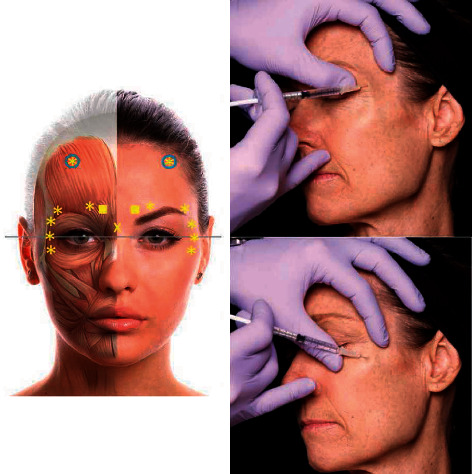
Botox was injected into five eyebrow areas and eight crow's feet (four on each side) for eyebrow lift. The symbols represent different injection depths: the squares represent the full depth of the needle; X represents half needle depth; asterisk indicates one-third of the needle depth [51].

Botulinum toxin is most commonly used to treat the upper part of the face to eliminate or reduce frown lines (prebrow and wrinkle muscles), forehead lines (frontal muscles), and periorbital or crow's feet lines (lateral to the orbicularis oculi muscle). Common therapeutic muscles include the frontalis, procerus, corrugators, and orbicularis oculi.  Figure 2 shows the injection of botulinum toxin into the five glabella sites and 3-4 sites on each side of the face. The use of short, small-bore needles can help minimize the trauma. In the middle part of the face, the reduction in the volume of facial fat and sagging caused by gravity are the main causes of aging [47]. In this part, it is limited for botulinum toxin to treat facial aging, whereas fillers are more commonly used. Treatment of the lower part of the face is usually focused on the perioral wrinkles (orbicularis oris, proximalis, and mentalis muscles) and requires greater skill and small doses.

Generally, a combination of such treatments can produce better results. Botulinum toxin injection into the lower part of the orbicularis oculi muscle, static crow's feet, and deeper frontal lines combined with skin peeling therapy, such as laser or chemical peeling therapy, can improve the efficacy of static lower eyelid wrinkles. Botulinum toxin is better in raising the lateral eyebrows and treating static lip lines in combination with dermal filler injections.

#### 3.1.2. Contraindications

Patients with neuromuscular diseases, such as myasthenia gravis, Eton–Lambert syndrome, and multiple sclerosis, are unsuitable for botox injections [52]. Additionally, it is not suitable for women who are menstruating and pregnant. It is also not suitable for people who are allergic to albumin and botulinum toxin. It is not recommended for patients with a blood disease or abnormal coagulation function; severe diabetes; heart, liver, kidney, or lung diseases; and severe hypertension. It is not appropriate in emaciated people because the muscle is too thin. Furthermore, the injection can easily spread to the surrounding muscles, thus resulting in side effects.

#### 3.1.3. Complications

Treatment with botulinum toxin is safe. Most adverse reactions to botulinum toxin are usually due to exaggerated action of the drug and its spread to unintended areas, often with significant or disfiguring complications. One of the commonest complications is posttreatment facial drooping [53]. For example, the most important complications in the glabellar complex and forehead area are drooping of the upper eyelid and eyebrows. In the treatment of crow's feet, an improper injection may result in diplopia, incomplete closure of the eye, lateral drooping of the lower eyelid, and asymmetrical smile when injected into the zygomaticus major muscle [52]. It has also been noted that, after treatment for wrinkles around the lips, some patients reported feeling abnormal after speaking; however, this situation resolved after multiple treatments [54].

It is important to note that normal reactions after such treatments are not true complications; however, needle marks, swelling, bruising, and early discomfort are generally considered complications by the patients. Many patients consider the normal response after the treatment to be abnormal, which may be related to their expectations.

## 4. Facial Fillers

The main facial filler materials include hyaluronic acid, calcium hydroxyapatite, poly-L-lactic acid, collagen, autologous fat, and polymethyl methacrylate. Different types of facial filler products are used in different fields of microinvasive surgery for facial rejuvenation. Each of them offers unique advantages and disadvantages (Table 2) and can solve different aspects of facial aging, such as the loss of collagen, fat displacement, and hypertrophy.

### 4.1. Hyaluronic Acid

In 1934, Carl Meyer and his assistant John Palmer separated a newly discovered glycosaminoglycan from the glass of a bull's eye and named this substance “hyaluronic acid” [73]. Hyaluronic acid is a natural linear polysaccharide polymer consisting of repeated diglucan units of N-acetyl-D-glucosaminoglucose and D-glucuronic acid linked by *β* (1, 4) as well as *β* (1, 3) glycosidic bonds [74]. It differs from other glycosaminoglycans as it lacks sulfated groups and covalently linked peptides [75]. Hyaluronic acid is an integral component of the extracellular matrix in most mature organisms and is found naturally within most body tissues, including the skin [76]. In addition, hyaluronic acid has been used extensively in the treatment of various conditions, such as infrabony periodontal defects. [77, 78].

Although hyaluronic acid may undergo volume changes due to natural decomposition processes, it has a relatively long shelf-life [79, 80]. It also attracts moisture into the skin [81]. Hyaluronic acid injections can, therefore, be used to replace the loss of volume on the face to restore its youthful appearance [82]. Most hyaluronic acid products are injected by mixing powdered hyaluronic acid in a particular ratio with water to produce a solution. There are two main sources of hyaluronic acid used in facial injections: animal-derived and non-animal-derived sources. Animal-origin hyaluronic acid is obtained from the crowns of chickens, which may also be responsible for the rare allergic reactions in avian animals [83]. Nonanimal sources of hyaluronic acid are of bacterial origin. Such hyaluronic acid may contain very small amounts of bacterial proteins, which may also trigger an allergic reaction [84, 85].

#### 4.1.1. Indications

**Figure 3:**
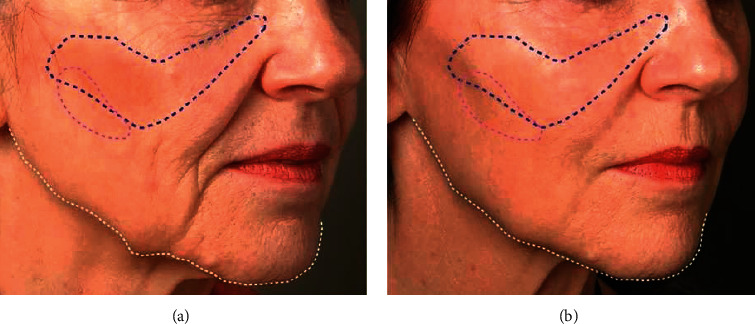
(a) 67 year-old patient shows multiple signs of aging due to loosening of the soft tissues in several areas, such as sagging in the mid and lower face, and resorption of the upper and lower jaws. (b). The patient's face 10 months after hyaluronic acid injection. No surgery was performed. The changes are due to providing prominence or lift, restoring the structure and volume to the cheek mound and mid-cheek (marked in blue), weakening the shadows of the cheeks (marked in purple), and repairing the borders of the mandible (marked in white) [35].

Skin wrinkles caused by the loss of collagen and elastin fibers in the fine dermis can be effectively addressed using hyaluronic acid fillers [3]. The increasing sophistication and varieties of modern hyaluronic acid injections have made injectable fillers an appropriate intervention to address various aspects of facial aging, such as contouring, balance, and feature positioning, rather than just diminishing the skin wrinkles (Figure 3) [86]. Generally, a person's lips shrink and wrinkle with age. Filling the lips with hyaluronic acid can achieve lip volume restoration and contouring [41, 86]. Facial aging causes variations in the distribution of subcutaneous tissue, particularly around the temporal area, orbits, cheeks, and corners of the mouth [42]. The appearance of lines can result in an aged look of the face [87]. Hyaluronic acid is very popular among beauty seekers because of its good performance as a filler. It can also be used to fill scars caused by trauma and surgery, asymmetries resulting from congenital defects, and pits from acne scars [35]. Hyaluronic acid injections are administered in rhinoplasty. This method includes the advantages of quick shaping, no surgery, and a painless procedure [88]. Hyaluronic acid is injected into the forehead where it fuses with the pre-existing hyaluronic acid, thus producing a skin swelling and forehead augmentation effect [89]. It is noted that a physician's knowledge of hyaluronic acid's specific properties in terms of rheology is crucial in selecting the appropriate hyaluronic acid products as well as determining the facial areas eligible for their use [90].

#### 4.1.2. Contraindications

Patients with past medical experience with cosmetic surgery for rhinoplasty are at risk of skin necrosis [91]. Several factors may contribute to the risk of skin ischemia, gangrene, and vascular embolism after hyaluronic acid injections, such as the unpredictability of vascular localization [92].

#### 4.1.3. Complications

Acute complications with hyaluronic acid injections are rare; they include nerve damage, pain due to venous and lymphatic injury, severe bruising due to vascular injury, partial pressure necrosis of the skin, and distal soft tissue necrosis, which can have serious functional and esthetic implications [93, 94]. Hyaluronic acid carries a very low risk of allergic reactions; therefore, preprocedural skin testing is hardly required [95, 96]. Redness and swelling are common side effects of filler injections. They usually have no sequelae but can lead to temporary local bleaching of the skin that has responded to manipulation and hyaluronidase and potential scarring. Additionally, there may be bruising, nodulation, and irregularities [97]. If hyaluronic acid is carelessly injected into a blood vessel, it may lead to necrosis, delayed reticular erythema, and pain in distant skin [98]. If the arterial blood flow is connected to the ophthalmic system, it may lead to retrograde retinal arteries, ocular muscle paralysis, or even unilateral blindness [99, 100]. Activation of herpes may occur after hyaluronic acid injection with symptoms of erythema and crusted papules that require antiviral treatment with acyclovir. Although this is extremely rare, it should be taken seriously by practitioners to ensure proper prevention and timely diagnosis and treatment [101]. When administered by a board-certified expert dermatologist, hyaluronic acid is extremely safe as an injection, and the incidence of side effects is extremely low [102].

### 4.2. Calcium Hydroxyapatite

Compared with other facial fillers, calcium hydroxyapatite plays a unique role in increasing action time and filling effect. It is the main mineral in human bones and teeth; therefore, it has good biocompatibility with the human body. Radiesse® (Merz Aesthetics, New York, USA) is composed of hydroxyapatite calcium microspheres of 25–45 mm suspended in water containing glycerol and carboxymethyl cellulose-containing hydrogels. As calcium hydroxyapatite is utilized immediately for the repair of facial appearance, the residual microspheres form a scaffold for the growth of fibroblasts. Additionally, the collagen fibers that are formed can fix the microspheres and prevent them from moving. These microspheres dissolve in a piecemeal manner into calcium and phosphorus ions within a few months to a year [57]. In 2011, Radiesse® was approved by the FDA for the treatment of facial fat atrophy in patients with infection with human immunodeficiency virus (HIV) and the treatment of moderate-to-severe facial wrinkles. Compared with hyaluronic acid, calcium hydroxyapatite has a higher elastic modulus [58].

#### 4.2.1. Indications

There is no doubt that Radiesse® can be used for filling soft tissues. [58] Additionally, it is now more commonly used to stimulate collagen production within the facial skin to improve the quality of the skin [59]. One periorbital treatment uses calcium hydroxyapatite to treat pigmentation caused by lacrimal duct malformation [60]. Radiesse® has been used in treating the puppet line, premaxillary groove, oral commissure, and posterior mandible. It is reported that some people believe that when injected into the temple and zygomatic area, it will also produce positive clinical effects [58].

#### 4.2.2. Contraindications

Although hydroxyapatite calcium has become the second most popular filling agent after hyaluronic acid due to its short marketing time and good biocompatibility with the human body, its use is limited to the lacrimal groove area of the lip and the lower orbital margin. Additionally, injections should be avoided at sites of inflammation [12].

#### 4.2.3. Complications

The most common postinjection side effects are usually limited to 2 weeks and include erythema, ecchymosis, and edema. Tzikas et al. [103] examined 1000 patients who were injected with calcium hydroxyapatite and noted only minor side effects of bruising, redness, and itching. The incidence of nodule formation was 5.9% in the lip and 0.002% in other parts. Improper injection methods may lead to the formation of nodules; therefore, lip injections should be avoided. If it is injected at a site of inflammation, it will worsen the inflammation further. It can also cause long-term swelling and bruising which can be avoided by selecting the appropriate technique and method of injection [12]. Rare complications of these injections include palpable vascular occlusion and nodules. Larger nodules can be injected with lidocaine and 5-fluorouracil to reduce the fibroblast activity and break them down [58].

### 4.3. Poly-L-Lactic Acid

**Figure 4:**
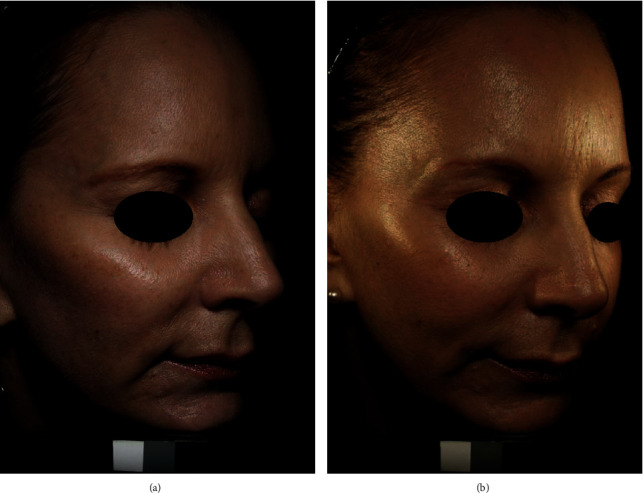
(a). Before Sculptra (Galderma, Fort Worth, TX, USA) injection. (b). 13 months after PLLA. Each of temporal, midface, and submalar areas received two vials of Sculptra [102].

Poly-L-lactic acid (PLLA) is a biocompatible, biodegradable, *α*-hydroxyl-based synthetic polymer, which has been used clinically as the main component of some surgical absorbable sutures for over 20 years [58]. A PLLA filling layer is placed within the deep dermis or subcutaneous layer. PLLA microparticles are large enough to avoid phagocytosis and immediately result in subclinical foreign body inflammation, which leads to encapsulation of the microparticles, fibrous tissue hyperplasia, and type I collagen deposition in the extracellular matrix to achieve the esthetic effect of filling. Furthermore, some studies have reported the presence of type III collagen near the PLLA microparticles [66]. After the injection, the volume of the patient's face may increase immediately due to mechanical expansion of the particle suspension, which settles within a few hours to days. The level of expansion is an approximation of the overall results after three treatments; therefore, it can be used to predict the number of treatments needed to reach the expected results [66]. Having received the injection, the initial effects appear gradually after one month and are more remarkable over 3–6 months (Figure 4). PLLA particles are metabolized by the same metabolic pathway as that of lactic acid; they reduce by 6%, 32%, and 58% at 1, 3, and 6 months, respectively, and degrade completely by approximately 9 months [66].

The effects of PLLA are long-lasting and can achieve effect that lasts for 2 years after three consecutive sessions. Skin tests can be avoided for sources that are made by nonhumans or nonanimals. PLLA can also be applied to deep tissues and around the bones. However, drawbacks of PLLA include typical features of injectable dermatological agents, such as ecchymosis, transient pain, mild-to-moderate hematomas, inflammatory response, edema that lasts for 3–5 days, irregular appearance (depending on the injection technique), and multiple treatments. The FDA approved PLLA as the only injectable implant for correcting HIV-associated facial fat atrophy in 2004 but also introduced it for cosmetic uses, including facial wrinkles, nasolabial creases, and other facial contours [104].

#### 4.3.1. Indications

PLLA can be used in patients with or without HIV infection (including retroviral treatment) with facial lipoatrophy; they will need sufficient patience, however, due to the time required for the gradual effects of PLLA to manifest. The applicable parts include the zygomatic and cheek depressions, lower facial relaxation and fold, lower orbital depression, neck lines (venus chain), and hands.

#### 4.3.2. Contraindications

PLLA is contraindicated in patients with collagen allergy, related immune system diseases, scar hyperplasia, hematologic diseases, and coagulation disorders during pregnancy and lactation. Orbital infarction with loss of vision has been reported after unexpected intravascular PLLA injections around the nasal and periorbital areas [104]. Treatments should be avoided over the nose, suborbital areas, and lips as these areas are at higher risk of overcorrection, nodules, and intravascular injections that are attributed to continual muscle contractions [104].

#### 4.3.3. Complications

PLLA has been used for a long time with a good safety record. Common complications are generally mild and mostly resolve spontaneously. Granuloma and nodular formation are major complications with PLLA. They occur occasionally and are related to incorrect injection areas and techniques. Postoperative swelling, redness, pain, itching, discoloration, scab formation, and peeling are common phenomena. As long as a reasonable dosage is injected, these symptoms resolve spontaneously within a few days. Some patients can have an acne outbreak after treatment for 10 days, which is related to abnormal stimulation leading to vigorous metabolism. It is suggested that PLLA should be injected into the deep or underlying dermis to establish the supporting structures. If the injection level is too shallow, skin nodules and/or fever are likely. Skin nodules can easily appear, if the dose or concentration is too large or the preparation time is insufficient.

Dermal thickening is slow and progressive. Therefore, according to the principle of “a small number of times,” which has been designed to strictly prohibit “overcorrection,” there should be 3-4 weeks of observation between two injections. According to the degree of tissue atrophy, usually, 3-4 continuous treatments are required to achieve the most satisfactory effects. Standard volumetric solutions require a maximum of four treatments 4–6 weeks apart [58]. Some researchers also recommend higher reconstitution volumes (5–9 mL with a maximum of 10 mL) and longer hydration times (24–48 h). All these techniques have been demonstrated to be effective in reducing the incidence of nodules [58, 66]. The injection areas should be deep and should never be used in areas with extremely thin skin, such as the lips and eyes. Therefore, comprehensive training is required for physicians. The correct injection method should be mastered, and its abuse must be avoided. PLLA injections should be avoided in areas where other fillers have been injected or sites of inflammation.

Ice compression is required immediately after the injection, and direct sunlight should be avoided as much as possible for half a month. After administering the injection, the areas must be gently massaged for even distribution of PLLA in the skin and minimize the formation of nodules and granulomas. The following massage principles should be followed: instruct patients to use over-the-counter petrolatum-based ointment 5 minutes at a time, five times a day, for 5 days to promote even distribution of the injected substance [58]. If there is a significant nodule or mass, it usually takes months or more to resolve spontaneously. If necessary, a small dose of corticosteroids can be injected for relief.

### 4.4. Collagen

Collagen, the main component of the dermal extracellular matrix, is an ideal biological scaffold that can provide space for fibroblasts' growth and is also a good medium for cellular growth. Injecting collagen into the human body results in not only the filling of valleys or wrinkles but also the induction of host cells and capillaries to migrate into the injected collagen. With adequate oxygen and nutrients delivered by capillaries, the host fibroblasts can undergo normal activities and synthesize the host's collagen and other extracellular stromal components.

**Figure 5:**
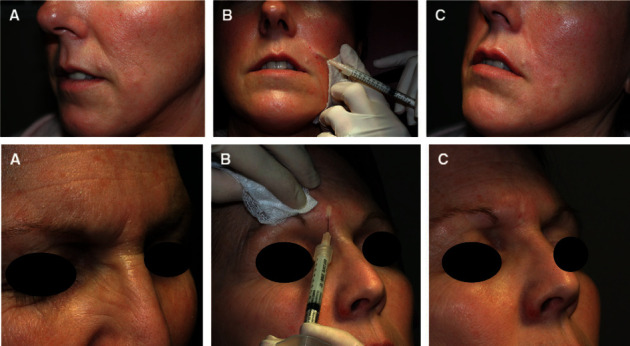
(a) Nasolabial fold fine lines and glabellar lines before collagen injection. (b) During collagen superficial injection, skin blanching is observed. (c) Immediately after collagen injection, remarkable results can be observed [105].

Collagen improves the skin's elasticity, increases water content, and reduces wrinkles (Figure 5). Collagen, one of the first injectable fillers used, has been used for more than 30 years and can be used alone or in combination with other facial fillers. The main collagen injectable products include bovine, human, and pig collagen products of different concentrations.

After 1-2 weeks of injection, collagen begins to mix with the host's collagen and is gradually absorbed and degraded by the skin tissue, making the skin feel increasingly natural. After 1-2 months, it will result in a softer and more natural feel than other types of fillers. Collagen does not easily absorb water and swell, so when used in tissues around the eyes that have slow water metabolism and can easily develop edema, there will subsequently be no water absorption and edema, which can reduce postoperative adverse reactions. When collagen is used for mesotherapy, the whitening and skin rejuvenation effects are stronger than those with hyaluronic acid. Collagen is an excellent coagulant with hemostatic effects, does not easily diffuse distally, and does not easily embolize (does not mean that it will not embolize). Therefore, it is relatively safe in areas with rich blood vessels, such as the eyelids and around the eyes, or in patients prone to bruising. Repeated injection of collagen can stimulate dermal fibroblasts to produce new collagen, form new tissues, and perform their functions of repair and regeneration.

Collagen injections can only be used against skin aging caused by superficial wrinkles, such as the small and shallow shrinkage skin defects of brow lines, nasolabial groove wrinkles, crow's feet, or superficial scars and brow lines. For deeper wrinkles, such as forehead wrinkles in elderly patients, and older or deep scars, such as those of cystic acne or postoperative scars, the effects are not perfect. Collagen has a short duration of action in the tissues, only 3–6 months, and continues to reduce in volume. Therefore, several injections are required, and the optimal time of satisfaction is short. Early collagen was derived from bovine or human cadavers, which are species-specific and tissue-specific. Certain people may develop allergic reactions; therefore, skin tests must be performed before its use. It may also have hidden dangers of animal-derived pathogens, such as mad cow disease. It must be stored in cold storage, which is inconvenient for transportation.

#### 4.4.1. Indications

The applications of collagen vary according to the filling level of different products and collagen compositions. The product that fills the dermo-epidermal junction is used for superficial skin lines (eyebrow lines, fine lines, crow's feet, scars, and horizontal forehead lines), while the product that contains more collagen is better suited for moderate and deep lines. Products with filling levels of the *epidermis* to the deep dermis are suitable for deeper wrinkles (nasolabial furrows, deep acne scars, and red lip edges) [67].

#### 4.4.2. Contraindications

Contraindications of bovine collagen and bioengineered human collagen include allergy to their ingredients, including lidocaine. Human cadaveric collagen is contraindicated in patients with gentamicin allergy, treated site infection, and collagen vascular disease. Autologous collagen has a low risk of hypersensitivity and is relatively safe [67].

#### 4.4.3. Complications

Complications are generally similar between the types of collagen injections. Common adverse reactions are injection overdose, irregularities, sclerosis, and unsatisfactory effects but are not extremely serious problems due to collagen's fast absorption rate. Therefore, serious complications associated with collagen products are rare. The commonest complication is hypersensitivity, which is commonly known as an allergy. Once serious allergies appear, the biggest advantage of collagen—that it can directly replenish the collagen in the skin leading to integration between the cells of collagen—becomes its most tragic shortcoming. Collagen injection products in the human body are very difficult to remove using operations and procedures, and they cannot dissolve as hyaluronic acid does; hence, such reactions are only managed with continuous allergy treatment to control the symptoms. Therefore, skin tests must be performed before using bovine and porcine collagen [105].

There are three types of collagen currently available:

Bovine collagen: bovine collagen products were the first soft tissue filling injections approved by the FDA. Before the development of hyaluronic acid, bovine collagen was often used as the gold standard for such injections [64, 67]. Bovine collagen is extracted from cowhide and has been used as a biomaterial for more than 30 years [62]. Most experts recommend that patients who receive bovine collagen injections undergo two skin tests 2–4 weeks apart [62]. Local allergy occurs in 3%–5% of patients. Adverse reactions to bovine collagen include allergy, bruising, local necrosis, bacterial infections, and reactivation of herpes virus infection [62].
Porcine collagen: following the development of bovine and human-derived bioengineered collagen, new pig-derived collagen appeared in the market. However, the data available on its use as an intradermal filler are scarce, and none are available in dermatological practice from the US. Porcine collagen is biodegradable and lasts for approximately 1 year in soft tissues. It has demonstrated good safety, is less immunogenic than bovine collagen, and has never resulted in allergic reactions. However, its main product, Evolence™ (ColBar LifeScience Ltd, Herzliya, Israel) was withdrawn from the American market by its manufacturer in 2009, just 1 year after it was approved [106].
Human-derived bioengineered collagen
  1. Bioengineered human collagen: cultured bioengineered human fibroblasts derive nutrition from the culture medium and produce collagen and extracellular matrix proteins, which can be used to manufacture the required face-filling implants. Pathogen screening is needed for fibroblasts, and the collagen they produce is affected by the suppression of the virus. The fibroblasts need to be screened for pathogens, and the collagen they produce is affected by viral inhibition. Allergy testing is not required because it is derived from human skin fibroblasts [62, 67].
  2. Human cadaveric collagen: this type of collagen product is an injection of a cell-free allogeneic dermal matrix tablet originating from human cadaver derma and marketed in 2000.
  3. Autologous collagen: the autologous collagen product is derived from a culture of autologous fibroblasts extracted from the skin of the patients. A 3 mm needle biopsy is performed, usually behind the ear, and sent to a laboratory for culture after being frozen. After approximately 6 weeks, 1–1.5 cm^3^ fibroblasts and extracellular matrix are usually obtained and returned to the doctor to be injected. Injections are to be administered within 24 hours of receipt of the product, and three injections with 2-week intervals are recommended [62, 67]. The disadvantages of the product are high costs, slow processing rates, painful injections, difficulties with immediate correction, and uncertain long-term efficacy [62].

### 4.5. Autologous Fat

Autologous fat has gained considerable popularity because it is an ideal filler with perfect biocompatibility. Autologous fat has many properties of an ideal filler and can be removed when necessary [107].

Autologous fat grafting was used at the beginning of the twentieth century to treat congenital deformities following oncologic surgery and has since been one of the procedures favored by plastic and maxillofacial surgeons [70, 107]. The clinical longevity of fat grafts, however, is highly uncontrollable; additionally, fat grafts have a high rate of absorption, and the results are operator-dependent [108]. A new technique was introduced by Coleman in the 1980s and is still the preferred method for fat filling and liposuction, which is constantly undergoing improvements [70, 71, 107].

Clinically, fat is often obtained from areas that have high fat content and no significant effects on the overall shape following aspiration. The inside of the thigh and lower abdomen are ideal supply sites because of the high concentrations of stromal vascular cells [108, 109]. To reduce pain and control bleeding, a swelling anesthetic technique is required. The swelling solution usually contains low concentrations of epinephrine, lidocaine, and saline.

**Figure 6:**
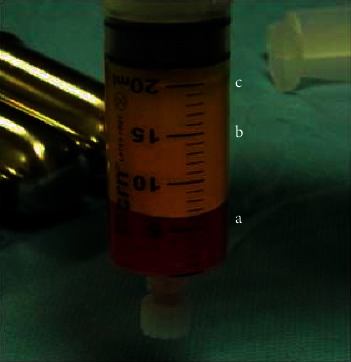
Centrifuged adipose tissue extract. From bottom to top: (a) First layer of blood and local anesthetics, (b) Second layer of fatty tissue, and (c) Third layer of lipids [62].

Fat processing is necessary because unfiltered fat can cause inflammation at the recipient site. Current mainstream techniques for fat processing include centrifugation, filtration, washing, and gravity settling (Figure 6). Each of these methods has its own drawbacks. Wang et al. reported that filtration and centrifugation techniques had better retention results [110, 111].

To improve the viability of fat cells, the fat tissue should be placed close to the blood supply during fat reimplantation. Multiple microinjections which are no more than 1 mL are preferable over a single injection [107].

#### 4.5.1. Indications

The main indication of facial autologous fat grafting is to correct facial contour deformities, such as craniofacial shortening, to eliminate scarring, wrinkles, and changes associated with aging. While the traditional approach relies on the removal of fat, mainly for subtraction, a more natural result is achieved by filling the hollow facial spaces. Fat grafting can fill the hollow spaces in the orbital and temple areas and improve the contours of the cheekbone areas and jaw.

The important injection areas include the lips, anterior jawline, and labial jawline. The deep “muscle-related” folds usually do not disappear completely with soft tissue enhancement. However, the texture of the fold can be improved [112]. Patients with mild brow ptosis and skin laxity can be rejuvenated using fat transfer. In these patients, fat grafting is often preferred [113].

#### 4.5.2. Complications

Lipotransfer is a very safe facial filler, and several trials have demonstrated that this method results in a good prognosis with few complications [114]. The commonest complication of autologous fat injections is fat necrosis that may be accompanied with hematoma and infection. Mild postoperative erythema and volume asymmetry have also been reported [110]. However, very rare and serious complications, such as blindness, sepsis, and hemiparesis, can also occur. Another study reported a rare case of ipsilateral external carotid artery (ECA) embolism following autologous facial fillers.

### 4.6. Polymethyl Methacrylate

Polymethyl methacrylate (PMMA) is a permanent filler. Its safety is similar to that of hyaluronic acid or calcium hydroxyapatite [115]. PMMA is very popular in facial filling because of its low price, easy availability, and simple usage [116].

The filling layer of PMMA is either the deep dermis or subcutaneous space. After injecting PMMA, the volume is initially provided by collagen, which is absorbed within 1–3 months. Meanwhile, the round, smooth PMMA microspheres are wrapped in the connective tissues of the host and are not degraded or excreted, thus becoming stable, permanent, and irreversible [115].

**Figure 7:**
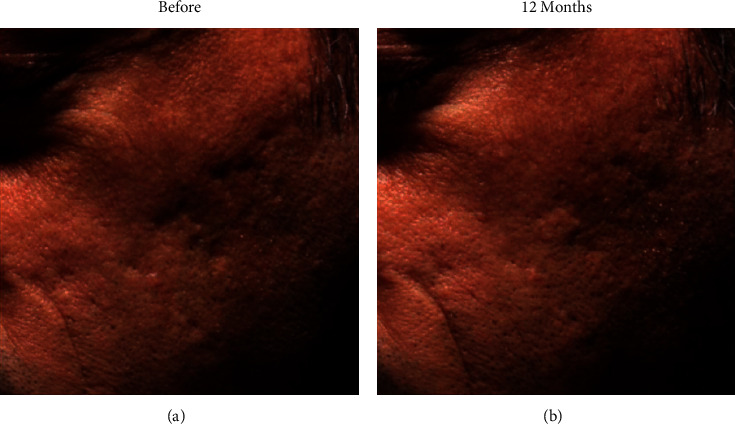
(a). Before and (b). 12 months after treatment of sectional scars using Bellafill® (Suneve Medical, San Diego, CA), significant recovery of scars can be observed in the cheek [115].

PMMA is not engulfed by human macrophages and does not undergo gradual degradation; therefore, its effects last for a long term or even are permanent (Figure 7). It can, however, ensure the softness of some tissues, and the effects are lasting in deep folds and acne scars. It is easy to implant but difficult to remove, and its nondegradability poses safety risks. It includes the risk of late granuloma and nodules that require steroid treatment or surgical resection. It has been marketed as a permanent skin filler in the US since 2007 and received FDA approval for treating acne scars in December 2014 [117].

#### 4.6.1. Indications

Injectable PMMA is suitable for the nasolabial groove as well as for the frown line, radial lip line, and corner of the mouth [72]. It can permanently resolve facial wrinkles, nasolabial creases, scars, and other skin defects.

#### 4.6.2. Contraindications

Complications of beading or masses have been reported with PMMA when injected around the eyes. If it is used for lip augmentation, it may result in a large number of nodules. Therefore, injections in these areas are not recommended [118].

#### 4.6.3. Complications

Immediate complications include ecchymosis and hematoma in patients who consume alcohol or are on antiplatelet aggregators, vitamin *E*, ticlopidine, and nonsteroidal anti-inflammatory drugs a few days before or after the implantation, which usually resolve spontaneously after 2–7 days [116]. For approximately 7 days after the implantation, most patients may develop swelling, which can be alleviated with ice and compression [116].

Infections can also result from an overdose of the injection and early local compression due to poor blood circulation. The potential risk of embolism cannot be excluded theoretically although it has not been reported. Rarely, advanced granuloma and tissue necrosis may also occur; the former occurs due to an allergic reaction to bovine collagen and PMMA, and the latter is mainly due to intravascular fillers or excessive compression of the surrounding blood vessels [116].

The late complications include the following. Approximately 6–24 months after implantation, PMMA microspheres may result in granuloma due to foreign body reactions, which can be treated with corticosteroid injections [116]. Many years after the implantation, migration of PMMA microspheres may occur because the particles are surrounded by collagen fibers and are often difficult to be absorbed by human body, which can present as sclerosis or nodules that require surgical interventions [116].

## 5. Thread Lift

As more patients opt for minimally invasive rejuvenation procedures, thread-lifting techniques have emerged as an excellent option. Thread lift has been used in medical esthetic surgery since the early 11010s [119]. Sulamanidze's Antiptosis (Aptos) Subdermal Suspension Thread hooks a special thread on the skin to make it tense, which counteracts the laxity of the tissue and finally achieves a cosmetic effect of eliminating wrinkles. Thread lift is popular because of its many advantages, such as smaller incisions and shorter recovery time [120]. Sasaki and Cohen originally used a hookless sharp thread for cheek fat pad lifts. However, its retention was not good; therefore, attention was turned to barbed sutures, which were named Aptos according to the number of barbs on the polypropylene threads [121]. A surgeon needs to have a good knowledge and understanding of the superficial musculoaponeurotic system (SMAS) because the threads are fixed underneath the skin tissue in the face and neck [122].

### 5.1. Types of Threads

#### 5.1.1. Aptos Threads

The Aptos thread is made of a cut 2-0 polypropylene thread. The thread has small, angled barbs in the same direction. Sulamanidze et al. [123] described the procedure as making a small incision in the temporary temple and drawing out several sutures under the skin. After the deeper threads and sutures appear, the remainder of the underlying line is clipped and the superior line is sewn to the temporalis muscle fasciatus using moderate traction. The recovery period with Aptos threads is short; however, patients need to follow their doctor's advice, such as avoiding sudden chewing and adhering to a massage for 1-2 weeks, to ensure that the results last longer. Indications for Aptos sutures in the face and neck are sagging, lax, flattened soft tissues, and less visible esthetic landmarks of the facial and cervical areas; lifting of the facial area is more common than lifting of the entire face. Examples include correction of significant nasolabial grooves or marionette wrinkles, lifting the cheeks, fixing the eyelids, and lifting fat from the lower eyelids [124]. The Aptos approach is considered by Sulamanidze et al. [125] to be the most effective in the midface region.

#### 5.1.2. Contour Threads

The contour thread is an isometric thread approved by the FDA in October 2004. It consists of 25 cm of 2-0 polypropylene suture with 50 intermediate 10 cm sections of unidirectional spiral barbs secured to the fascia. Carmina et al. [120] described its use as placing a 3 mm incision in the temporal hairline for upper and midface lifts and a surgical incision behind the latissimus dorsi muscle for a submental lift. The stitches are advanced in a ‘Z' shape in the subcutaneous plane as this shape is thought to maximize the number of hooks in touch with the subcutaneous tissues. The thread is finally truncated at the proximal end of the incision and secured to the deep fascia.

#### 5.1.3. Silhouette Sutures

The silhouette suture consists of junctions and tapers and is composed of approximately 82% PLLA and 18% poly (lactic-co-glycolic acid). The silhouette suture was approved by the FDA in April 2015 for use in draping midface tissue. The suture is conceived with 8, 12, or 16 tapers that are evenly spaced on either side within a 2 cm taper-free central area and are designed to reduce the risk of migration and extrusion. The cones are arranged in the opposite direction, and the tip of the cone points to the end of the suture. The degradable component of the sutures stimulates collagen production during the degradation process, which facilitates suture fixation. Silhouette sutures are indicated for moderate midfacial tissue declines, such as nasolabial folds, declining oral continuity, and marionette wrinkles. [126].

#### 5.1.4. Multianchor Suspension Sutures

Multianchor suspension suture is made from a 3-0 polypropylene suture. It has nine knots in the middle and spans 8 cm (knots are approximately 10 mm apart). Every knot is inset into an assimilable hollow cylinder made of copolymers containing levulinic acid and acetyl cross-esters. The sutures have a semicircular pin at the near terminus, which allows them to be fixed to a nonabsorbable synthetic knitted surgical mesh during the procedure. Multianchor suspension sutures are often indicated to lift the midface, for example, to decrease the width of the nasolabial groove, increase the definition of the mandible, and increase the temporal projection [127]. The technique can also be useful in static correction of nerve palsy of the face when recovery of the neurological functions is impossible.

### 5.2. Complications

The most common complications are the same as those with most logical surgical procedures, such as pain, swelling, and bruising. The most common complications are temporary. Additionally, depression of the skin and breakage or migration of the threads may also occur [120]. The incidence of nerve damage is lower than that of conventional debridement (0.7%–2.5%) [128]. Persistence of foreign objects can lead to many side effects as well as unsatisfactory esthetic effects [129]. Wu et al. [130] have demonstrated that complications, such as infection or granulomas, can also occur. Due to tissue remodeling, there is an increased likelihood of scar formation, hyperpigmentation, and suture extrusion.

## 6. Conclusion

Minimally invasive facial surgery mainly uses drug injections, fillers, thread lift, and radiofrequency to improve facial function and appearance. Compared with traditional cosmetic surgery, such as face-lifting, non-surgical modalities is increasingly favored by cosmetic surgeons and patients because of its advantages, such as small wound surface, short recovery period, and more natural effects.

Facial aging is mainly caused by the breakdown of collagen, elastin, and glycosaminoglycans, run-off of fat volume and displacement of fat in different locations, changes in muscle tension and length, and changes in the shape of bones. The different parts of the face experience different levels and likelihood of aging. In this article, the authors have listed the commonest areas of facial aging.

This paper summarizes the injectable drugs (botulinum toxin) and facial fillers (hyaluronic acid, calcium hydroxyapatite PLLA, collagen, autologous fat, and PMMA) in the treatment of facial aging along with their mechanisms, indications, contraindications, and complications as well as introduces thread lift and radiofrequency anti-wrinkle applications in treating facial aging. Botulinum toxin blocks cholinergic nerve transmission and causes muscle paralysis and relaxation, thus reducing hyperactivity of the muscles of expression and eliminating wrinkles. The main purpose of facial implants is to plump the face. Hyaluronic acid is naturally present in body tissues, including the skin, and has good compatibility with the human body. It is the most widely used facial filler currently, and the application range of calcium hydroxyapatite is second only to that of hyaluronic acid. The main action place of the thread lift is the SMAS. The cosmetic effects of fading wrinkles are achieved by hooking special threads under the skin to make it taut. The role of radiofrequency rhytidectomy removal is to heat the dermis and stimulate the regeneration of collagen so that the skin recovers its elasticity gradually and skin wrinkles are eliminated.

Although the mechanism of action of various drug materials is relatively clear, the indications, contraindications, and complications of their effects remain unclear for some clinicians. The comprehensive summary of injectable drugs, facial fillers, thread lift, and radiofrequency rhytidectomy presented in this paper can act as a reference for clinicians.

The combined application of facial nonsurgical modalities has good clinical prospects. Additionally, minimally invasive rhinoplasty, lip surgery, and lower eyelid lifting are becoming increasingly popular among patients.

## Figures and Tables

**Table 1:** Botulinum toxin injectable products.

Trade name	Toxin component	Molecular weight	Approved by FDA	Storage time
Botox	OnabotulinumtoxinA	900 kDa	+	>2 weeks
Dysport	AbobotulinumtoxinA	500/900 kDa	+	>2 weeks
Xeomin	IncobotulinumtoxinA	150 kDa	+	4 years

Information from references. [45, 47].

**Table 2:** Major facial filler materials.

Filler product	Type	Composition	Allergy test required	Indication	Advantages	Disadvantages	
Perlane® (medicis, montreal, CA)	HA (bacteria)	Hyaluronic acid (8000 gel Particles/mL), 0.3% lidocaine	No	Deep layer	Long-lasting effects, suitable for use in deep tissue and paracone, minimal reactions	Heavy edema; persists for 3–5 days	
Restylane® (medicis, montreal, CA)	HA (bacteria)	Hyaluronic acid (100,000 gel Particles/mL)	No	Superficial layer	Long-lasting effects, suitable for use in deep tissue and paracone, minimal reactions	Heavy edema; persists for 3–5 days [55]	
Restylane fine lines® (medicis, montreal, CA)	HA (bacteria)	Hyaluronic acid (200,000 gel Particles/mL),0.3% lidocaine	No	Dermo-epidermal junction layer	Particularly suitable for the area between the eyebrows and for fine lines, not suitable for parabones	Shorter duration than restylane or perlane [56]	
Radiesse® [57] (medicis, montreal, CA)	CaHA	Hydroxyapatite calcium microspheres, water, glycerin, and carboxymethyl cellulose	Yes	Facial soft tissue filling [58] stimulates collagen production in facial skin [59] pigmentation due to malformation of the lacrimal passage [60]	Good compatibility with the human body compared with hyaluronic acid, it has a greater modulus of elasticity [58]	Easy degradation and short maintenance time	
Sculptra® (galderma, fort worth, TX)	PLLA	150 mg of PLLA microparticles, sodium, carboxymethylcellulose and non-pyrogenic mannitol [56]	No	Zygomatic and cheek depression, lower facial relaxation and fold, lower orbital depression, neck lines (venus chain) and hand aging	Long-term results, safe, no allergy test needed [61]	Inflammatory response and edema last for a long time (3–5 days), irregular appearance (depending on injection technique), requires multiple treatments	
ZydermI® (INAMED, santa barbara, CA)	Bovine collagen	3.5% bovine dermal collagen suspended in physiological phosphate buffer sodium chloride solution and lidocaine [62]	Yes	Superficial skin lines [63] (eyebrow lines, horizontal forehead lines, crow's feet, fine lines, and scars)	Safe, reliable, contains lidocaine (lidocaine inhibits eosinophilic activation and reduces the risk of swelling and crusts), ease of administration [64]	Requires allergy test, more inflammatory response, short duration	
Zyderm II® (INAMED, santa barbara, CA)	Bovine collagen	6.5% bovine dermal collagen, suspended in physiological phosphate buffer sodium chloride solution and 0.3% lidocaine [62]	Yes	Moderate-to-deep wrinkles	Same as zyderm I	Same as zyderm I	
Zyplast® (INAMED, santa barbara, CA)	Bovine collagen	Cross-linked with 0.0075% glutaraldehyde, other ingredients are the same as in ZydermI [62]	Yes	Deeper wrinkles [63] (nasolabial furrows, deep acne scars, and red lip edges)	Same as zyderm I but more viscous and harder to degrade [64]	Same as zyderm I	
CosmoDerm I® (INAMED, santa barbara, CA)	Bioengineered human collagen	3.5% collagen, phosphate buffer saline, and lidocaine [65]	No	Superficial wrinkles [65]	Safe, reliable, contains lidocaine (lidocaine inhibits eosinophilic activation and reduces the risk of swelling and crusts), no allergy test needed, ease of administration [64]	Short duration
CosmoDerm II® (INAMED, santa barbara, CA)	Bioengineered human collagen	6.5% collagen, phosphate buffer saline, and lidocaine [66]	No	Same as CosmoDerm I [65]	Same as CosmoDerm I [65]	Same as CosmoDerm I
CosmodPlast® (INAMED, santa barbara, CA)	Bioengineered human collagen	Cross-linked with 0.0075% glutaraldehyde, other ingredients are the same as in CosmoDerm I	No	Medium-depth wrinkles and red lip edges	Same as CosmoDerm I, more viscous and harder to degrade [64]	Same as CosmoDerm I
Cymetra® (INAMED, santa barbara, CA)	Human cadaveric collagen	Micropowdered cadaver collagen [67]	No	Lip, nasolabial groove, and deep wrinkles [67]	Safe, contains lidocaine (lidocaine inhibits eosinophilic activation and reduces the risk of swelling and crusts), no allergy test needed	Skin necrosis if used in glabella, expensive, and often lumps in the needle
Isologen (isologen technologies inc., paramus, NJ)	Autologous collagen	A culture of autologous fibroblasts extracted from the patient's own skin [68]	No	Moderate-to-deep wrinkles	Safe, no allergy test needed	High cost, long processing time, injection pain, difficulty in immediate correction, and uncertain long-term efficacy [62,68]
Autologous fat	Autologous fat	Glycerol and fatty acids [69]	No	Facial contour deformities, scarring, and wrinkles [70]	Highest biocompatibility [71]	High resorption rate [71]
Bellafill® (suneve medical, San Diego, CA)	PMMA	Smooth microsphere of PMMA (20% volume) and 3.5% bovine collagen (80% volume) and 0.3% lidocaine matrix suspension [72]	Yes	Nasolabial groove, frown line, radial lip line, and the corners of the mouth [72]	Long-term effects or even permanent [65]	Requires allergy test, non-degradable components have safety risks [61]

HA, hyaluronic acid; CaHA, calcium hydroxyapatite; PLLA, poly-L-lactic acid; PMMA, polymethyl methacrylate.

## Data Availability

All data, figures, and tables in this review paper are labeled with references.
